# Immunoglobulin Structure Exhibits Control over CDR Motion

**DOI:** 10.4172/1745-7580.1000047

**Published:** 2011

**Authors:** Michael T. Zimmermann, Aris Skliros, Andrzej Kloczkowski, Robert L. Jernigan

**Affiliations:** 1 L. H. Baker Center for Bioinformatics and Biological Statistics, Iowa State University, Ames, IA 50011, USA; 2 Department of Biochemistry, Biophysics and Molecular Biology, Iowa State University, Ames, IA 50011, USA; 3 Bioinformatics and Computational Biology, Iowa State University, Ames, IA 50011, USA; 4 Battelle Center for Mathematical Medicine, The Research Institute at Nationwide Children’s Hospital, The Ohio State University College of Medicine, Columbus, OH 43205, USA; 5 Department of Pediatrics, The Ohio State University College of Medicine, Columbus, OH 43205, USA

## Abstract

Motions of the IgG structure are evaluated using normal mode analysis of an elastic network model to detect hinges, the dominance of low frequency modes, and the most important internal motions. One question we seek to answer is whether or not IgG hinge motions facilitate antigen binding. We also evaluate the protein crystal and packing effects on the experimental temperature factors and disorder predictions. We find that the effects of the protein environment on the crystallographic temperature factors may be misleading for evaluating specific functional motions of IgG. The extent of motion of the antigen binding domains is computed to show their large spatial sampling. We conclude that the IgG structure is specifically designed to facilitate large excursions of the antigen binding domains. Normal modes are shown as capable of computationally evaluating the hinge motions and the spatial sampling by the structure. The antigen binding loops and the major hinge appear to behave similarly to the rest of the structure when we consider the dominance of the low frequency modes and the extent of internal motion. The full IgG structure has a lower spectral dimension than individual F_ab_ domains, pointing to more efficient information transfer through the antibody than through each domain. This supports the claim that the IgG structure is specifically constructed to facilitate antigen binding by coupling motion of the antigen binding loops with the large scale hinge motions.

## Background

Immunoglobulin Gamma (IgG) is one of the principal players in the adaptive immune system and is commonly referred to as an antibody. It is produced in a huge array of diverse antigen binding forms by B-cells through a combinatorial process called V(D)J recombination. This produces molecules comprised of two heavy and two light chains with highly variable complementary determining regions CDRs. The heavy chains of a given antibody are identical in sequence to one another and are comprised of four immunoglobin folds (a two layer sandwich of 7 anti-parallel beta strands), one of which is variable in sequence. Light chains are also identical in sequence and consist of two immunoglobin folds, one of which is variable. The two light chain and the first two heavy chain (including the variable) immunoglobin domains come together to form an antigen binding domain (one for each pair). The remaining four (two per chain) heavy chain immunoglobin folds interact with each other to form a third domain that is often referred to as the constant region. IgG has been studied by proteolysis which cuts the structure into the three described fragments. For this reason the two types of domains which comprise the IgG are referred to as the antigen binding fragments, or F_ab_, and the constant fragment, or F_c_. The region connecting the F_ab_ domains to the F_c_ is known to be a highly flexible hinge. This hinge region has 2–4 disulfide bonds bridging the heavy chains. Previously it has been excised from IgG and used in protein design as a molecular linker. This sequence has been extensively characterized and even synthesized [1,2]. While the primary characteristic of this region is its hinge flexibility, which was an impediment to resolving the structure early on, it has also been shown to have somewhat unique hydrophobic binding that allows it to be selectively bound to a stationary membrane so that either the F_ab_ or F_c_ fragments can be cut off with different proteases and recovered [3].

Here we compute a hinge map of IgG using Elastic Network Models (**ENM**s), show the extensive spatial freedom of the unrestrained F_ab_ domains that presumably facilitates binding, analyze the internal changes of the structure and how they affect the hypervariable CDRs, and apply a recently derived normal mode based kinematic simulation to generate motions of the structure. Motions of the structure are analyzed and a description of the high mobilities of the CDRs is provided.

## Materials and methods

### Elastic Network Model

To study the kinematics of IgG we use **NMA** (normal mode analysis) within the context of ENMs. Biological structures are often represented by C^α^ atoms connected with harmonic springs. This represents the protein structure as an elastic network. The Gaussian Network Model (**GNM**), the earliest and one of the simplest of the elastic network models, is used to compute the relative magnitudes of motion. It was originally proposed by Bahar, Haliloglu and Erman for coarse-grained models in 1997 [4,5], who applied the assumption postulated by Tirion [6] for atoms that both bonded and non-bonded contacts in proteins can be represented by a single universal spring. The Anisotropic Network Model (**ANM**) proposed in [7], can be used to compute the directions of motions of all points in the structure. We employ the ANM model throughout the present analyses. To generate an ANM model we first construct a Laplacian (or Kirchhoff) matrix using Equation 1 where *r_c_* is a cutoff radius (typically 10–13Å), *d_ij_* is the distance between atoms *i* and *j*, and γ is the spring constant. The potential energy of such a system is given by Equation 2. We then compute a matrix of second derivatives of the potential energy (see [7] for details), the eigenvectors (*Q_i_*) of which are called normal mode shapes, and the eigenvalues (ω_*i*_) are the corresponding squared frequency. For a given normal mode we can then compute fluctuations of the structure with Equation 3.

(1) $$\Gamma =\{\begin{array}{cc} -\gamma & d_{ij}\leq r_{c}\\ 0 & d_{ij}>r_{c}\\ -\sum_{k=1,k\neq i}^{N}\Gamma_{ik} & i=j \end{array}$$

(2) $$V=\frac{\gamma }{2}\Delta R^{T}\Gamma \Delta R$$

(3) $$\Delta R_{i}=Q_{i}\text{cos}(\omega_{i}t)$$

Extensive applications of NMA to biological and chemical systems have been discussed in Cui and Bahar [8], Jernigan and Kloczkowski [9], and Sen et al. [10]. Successes with these methods make it clear that functionally important motions of biomolecules are usually governed by packing density. These and many other studies have enabled computations of the important motions on time scales beyond the usual reach of atomic molecular dynamics (MD). ENMs can be generated for small and medium sized proteins in seconds or minutes; a huge gain in comparison to the extremely long computational times required for corresponding MD studies. Work by Bakan and Bahar suggests that ANM may even sample conformation space more thoroughly than classic MD [33]. It has been demonstrated that extremely large molecular assemblages can be even further coarse-grained without loss of the major important motions [11]. More detailed analyses are available by use of elastic models that employ mixed-resolution models, where most of the structure is coarse-grained but with the regions of special interest remaining in atomic detail.

The dominance of the low frequency normal modes is universal, and usually there are only a few of these characteristic motions that are truly important. Here we represent the mean-square fluctuations by using the lowest frequency non-zero modes.

### Kinematics of Proteins

Our method for solving the kinematics of coarse-grained protein structures is based on the Lagrangian equation for the potential and kinetic energy of the system, as described by Chirikjian and coworkers [12–16]. First, a rigid body translation and rotation of the structure is performed to place the origin of the coordinate system at the center of mass and so that the moment of inertia tensor is diagonal. The potential energy of the system of *N* points can then be written as in Equation 1. Note that Γ is the 3*N* dimension square stiffness matrix of the system.

The displacement vector of the system, ΔR(t) , could be calculated as

(4) $$\Delta R(t)=\sum_{i=1}^{3N}[\frac{1}{\sqrt{\omega_{i}}}\text{sin}(t\sqrt{\omega_{i}})Q_{i}Q_{i}^{T}\Delta \dot{R}(0)+\text{cos}(t\sqrt{\omega_{i}})Q_{i}Q_{i}^{T}\Delta R(0)]$$

This facilitates performing time-dependent kinematic simulations with the ENMs using any desired combination of normal modes fixed by the index *i* and by choosing appropriate phase angles to describe the displacements between the phases of the different normal modes.

### Computing Changes in Internal Distances

We also consider the displacements of the positions of points in the structure with ANM. The mean-square change in internal distance (**MSID**) is computed as

(5) $$<{\left(\Delta R_{i}-\Delta R_{j}\right)}^{2}>=<\Delta R_{i}^{2}>+<\Delta R_{j}^{2}>-2<\Delta R_{i}\cdot \Delta R_{j}>$$

These values are obtained directly from the inverse of the Hessian matrix, Γ , from which the normal modes are derived:

(6) $$<{\left(\Delta R_{i}-\Delta R_{j}\right)}^{2}>=(3k_{B}T/\gamma )*[\Gamma_{ii}^{-1}+\Gamma_{jj}^{-1}-2\Gamma_{ij}^{-1}]$$

where k_B_ is the Boltzmann constant, T absolute temperature, and γ the ANM spring constant. We can also consider the normalized change in internal distances. This metric can be used to compare the magnitude of internal distance changes.

(7) $$<{\left(\Delta R_{i}-\Delta R_{j}\right)}^{2}>\prime =\frac{<{\left(\Delta R_{i}-\Delta R_{j}\right)}^{2}>}{\sqrt{\Delta R_{i}^{2}}\sqrt{\Delta R_{j}^{2}}}$$

### Fractal and Spectral Dimension

As early as 1980 the fractal dimension of myoglobin was studied [17], and it was found to be about 1.65. Experimental analysis of the spectral dimension of lysozyme was recently performed [18]. This study revealed not only that proteins may exhibit a mix of phonon (exhibiting discrete vibrational modes) and fractal character but also that the spectral dimension is relatively low and shows only moderate sensitivity to temperature. This finding provides an explanation for the efficient information transfer through protein structures. More recently, Granek and Klafter showed mathematically that certain fractal structures (and not uniform lattices) will experience the type of autocorrelation decay that is observed in protein experiments [19]. The compactness of protein structures is represented by a fractal dimension between 2.3 and 2.7 (see Enright and Leitner [20]). Investigation of the spectral dimension of elastic networks and explaining its relation to real structures has also been carried out [21]. Spectral and fractal dimensions were related to one another in recent papers by Reuveni and colleagues [22,23]. An equation was proposed that relates the two dimension metrics that fits well with the 5794 surveyed protein structures [24]. Here, we utilize the methods described in Ref. [22] for calculating the spectral and fractal dimensions of IgG and relate these findings to the CDR motions.

The fractal dimension describes how the mass captured within concentric spheres scales with the radius of these spheres. It is calculated here by finding the ten points closest to the proteins center of mass. Concentric spheres with incremental radii of 1Å are constructed and the total mass captured within each is recorded. Linear regression is performed ten times, once for each of the points closest to the protein’s center. The average slope of the log-log plot of sphere radius versus mass captured is taken as the fractal dimension. The spectral dimension describes how the frequencies of vibration for the structure scale with the density of modes. That is, one performs a linear regression against the log-log plot of frequency versus the cumulative number of modes at each frequency. The spectral dimension is then the slope of this regression.

## Results and Discussion

We seek quantification of the motions of IgG in its dominant normal modes, which correspond to the flexing about the major hinge, particularly to see how this affects the spatial freedom of the CDR, both overall and internally.

**Figure 2:**
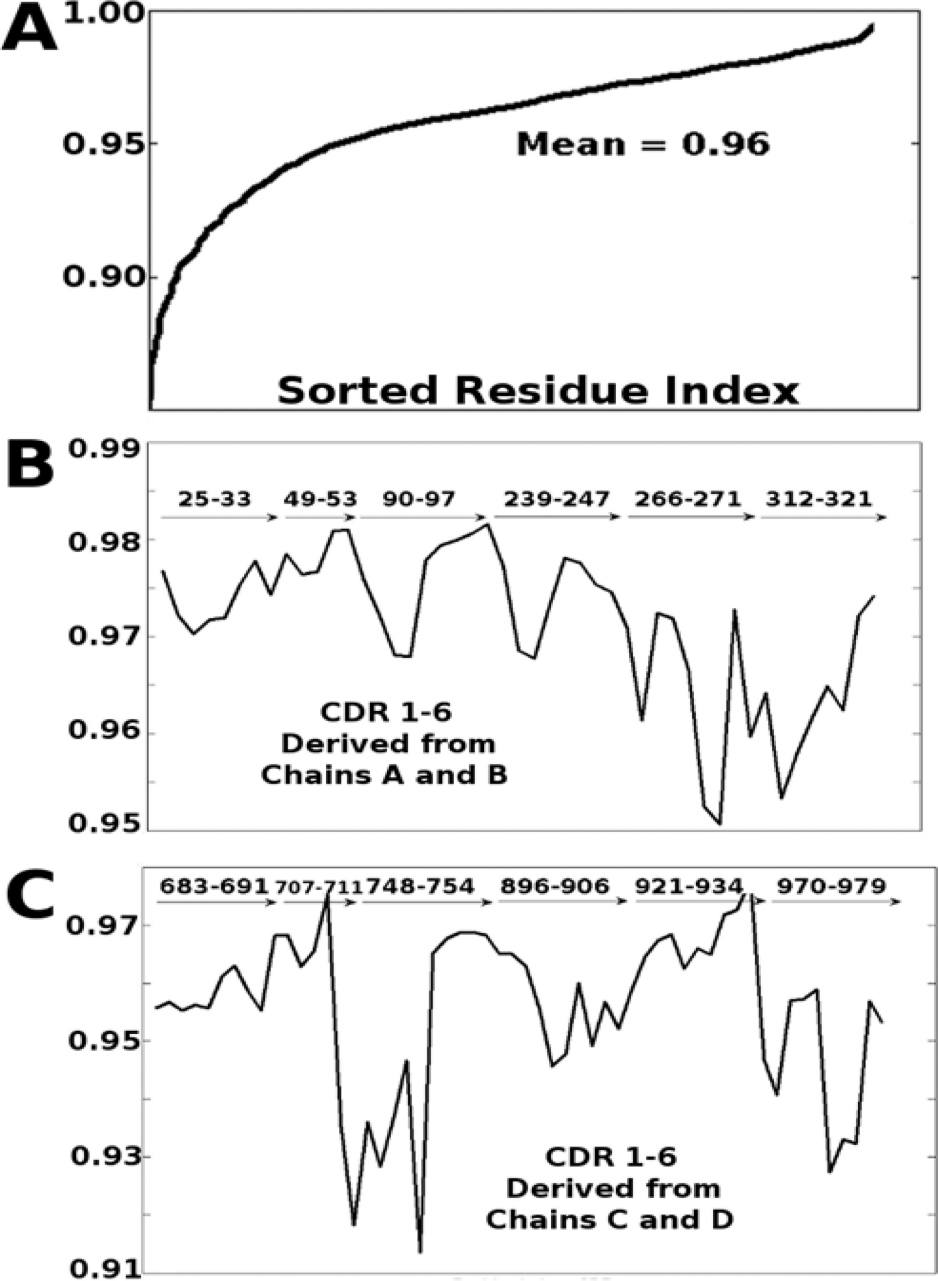
The first six modes of motion capture most of the total motion. Mean correlations of the motion derived from the first six normal modes with the total motion for (A) the entire structure, (B) the six CDR loops from chains A and B, (C) the six CDR loops from chains C and D. 1IGT has 1316 residues in total. The mean correlation over all residues is 0.96 showing that the slowest modes strongly dominate the intrinsic motions of the structure. Behaviors for each of the individual CDRs are shown.

Figure 2 shows the impact of the six slowest normal modes on the motion of the IgG and points out the CDRs. We see that these six normal modes account for nearly all of the motion of the Immunoglobulin, above 85% of the total motion for all residues and greater than 90% for the majority. This means that residues of the immunoglobulin move in a highly coordinated motion and that the loops do not act as in polymers, to randomly sample their dihedral angles. A large body of evidence shows that the ENM generates low modes that correspond with known biochemical functions of proteins. This gives us confidence to conclude that correlations of motion within a low frequency mode are pertinent to the function of the IgG.

We have also computed correlations between mean-square fluctuations calculated using only the six lowest frequency modes and using all modes, for all residues, and the CDR (see Figure 2). Similarly, we compare correlations computed by using all normal modes in equation 4 with those obtained by using only the slowest modes (see details in the Methods section).

**Figure 3:**
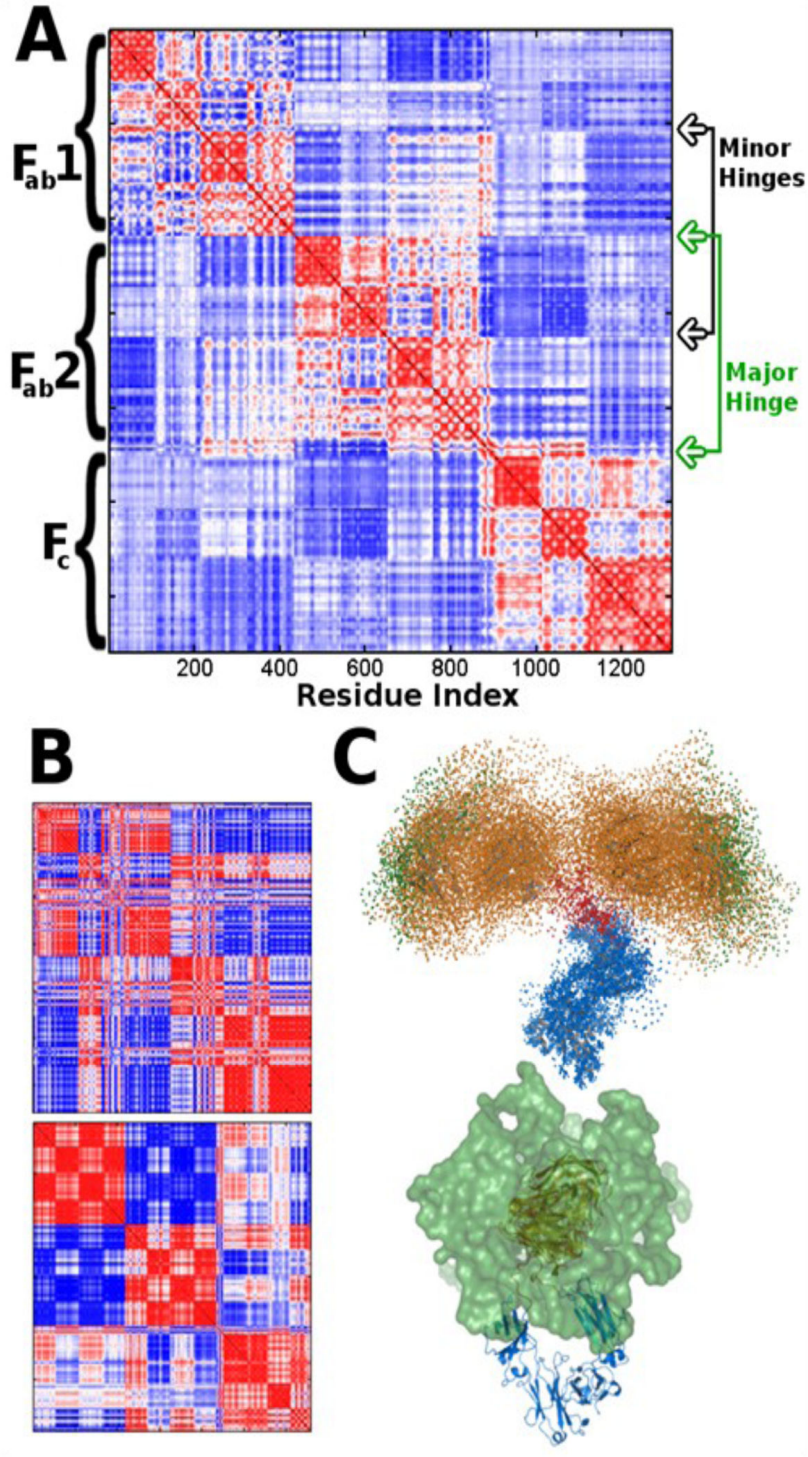
Hinges and the motion behaviors identified by computations. (A) Correlation matrices (dot products between normalized pairwise displacement vectors). All values fall in the range [1,−1]. The average over the first 9 modes is displayed, with red corresponding to the motion of C^α^ pairs positively correlated, blue for negative correlations, and white uncorrelated. From the block structure of the diagram and the changes in sign we can identify the hinge regions within the structure. (B) Similar to (A), but for individual modes; 1 (top) and 9 (bottom). (C) We use the 12 slowest modes to compute conformers of IgG because the majority of the motions in these modes are localized at the three prominent hinge regions. The F_c_ is aligned in all conformers. Structure coloring shows the F_c_ in blue, the major hinge in red, CDRs in green, and the remainder of the F_ab_ in orange (in two perpendicular views).

**Figure 4:**
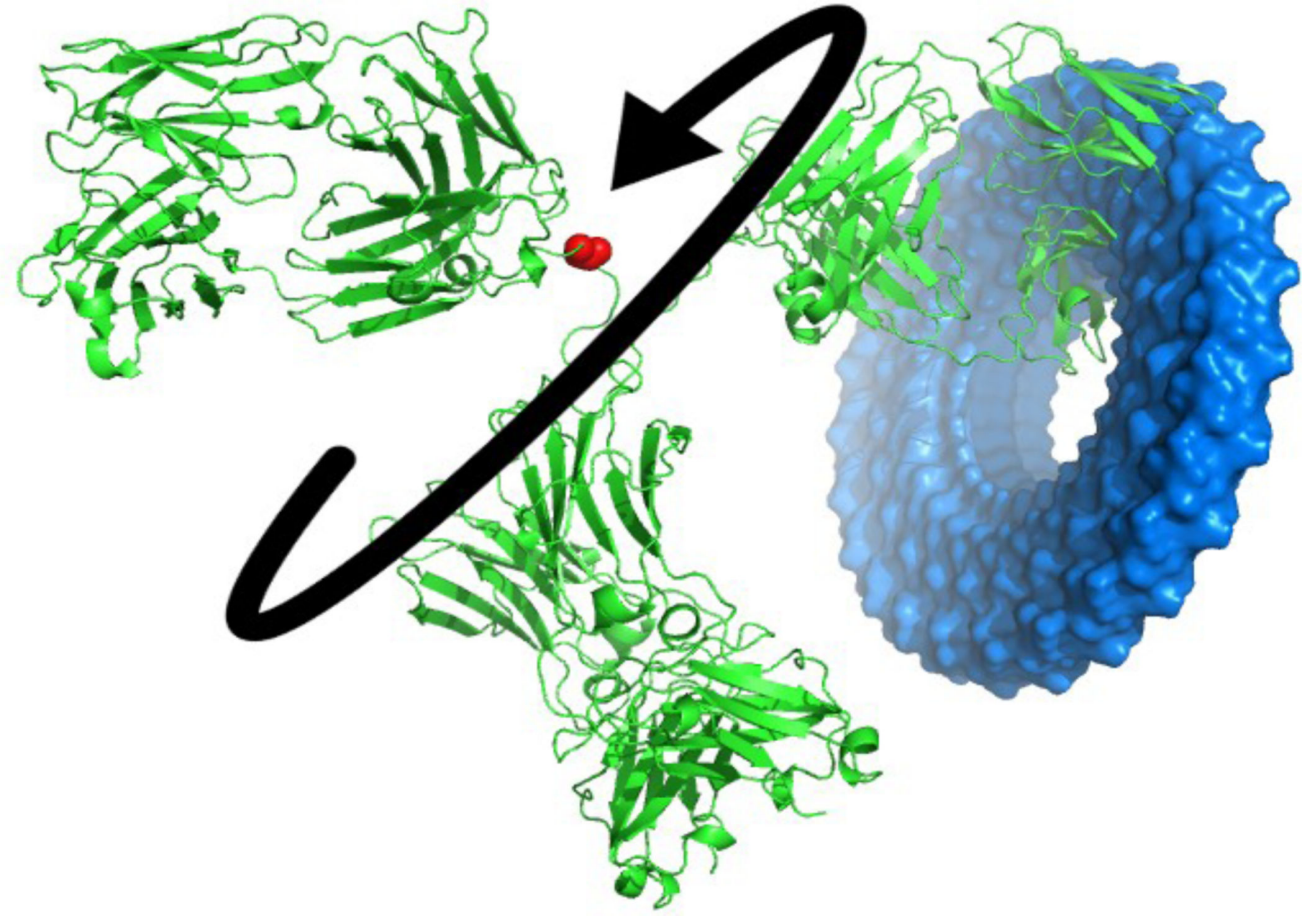
The sampling of hinge forms on the position of the CDR. In this figure the red amino acid’s psi-angle at the major hinge is varied in 15° increments and the resulting position of the CDR on the right side is accumulated. The collection of all of these CDR coordinates is shown as the blue volume similar to Figure 3(C). This visualization could be useful for IgG hinge analysis, but would require inclusion of the limitations imposed by torsion angle availability (Ramachandran space) and steric clashes.

Figure 2 shows that the mean impact of the first 6 normal modes on the total motion is about 96% and that the lowest frequency modes do provide an excellent representation of the overall motions of the system. From a visual inspection we see the low frequency modes are associated with domain motions, a behavior that is usually seen in multidomain structures. For this reason normal modes have been used to identify hinges within structures [25]. We perform similar computations to confirm the presence of the hinges within IgG (see Figure 3). In order to determine the extent of CDR sampling we generate conformers using the normal modes. The magnitude of deformation in each mode is set by choosing the largest deviation that does not substantially deform the sequential virtual bond lengths. The lowest frequency modes corresponding to the global motions are collective in nature and exhibit comparatively low virtual bond stretching. We find that overall the three domains are anti-correlated with one another. Figure 3 part C displays a representation of CDR sampling after following normal modes. It is apparent that the structure of IgG is designed to span the maximal space for the CDRs, presumably to aid binding. A similar approach for understanding the spatial freedom that the structure can sample would be to alter dihedral angles; Figure 4 illustrates this mapping approach, which is only preliminary. Other considerations would be required for more realistic dihedral sampling (steric hindrances, backbone constraints, energetic and inertial effects, etc.).Normal mode calculations are often performed to elucidate which residues or atoms in a molecular structure are the most mobile. Mobile active site residues may play roles in binding or substrate selection, whereas rigid regions are more likely to play key stabilization roles in the structure as a whole, as in a scaffold. An important exception to this occurs for the catalytic residues within an active site cleft that are relatively rigid.

**Figure 5:**
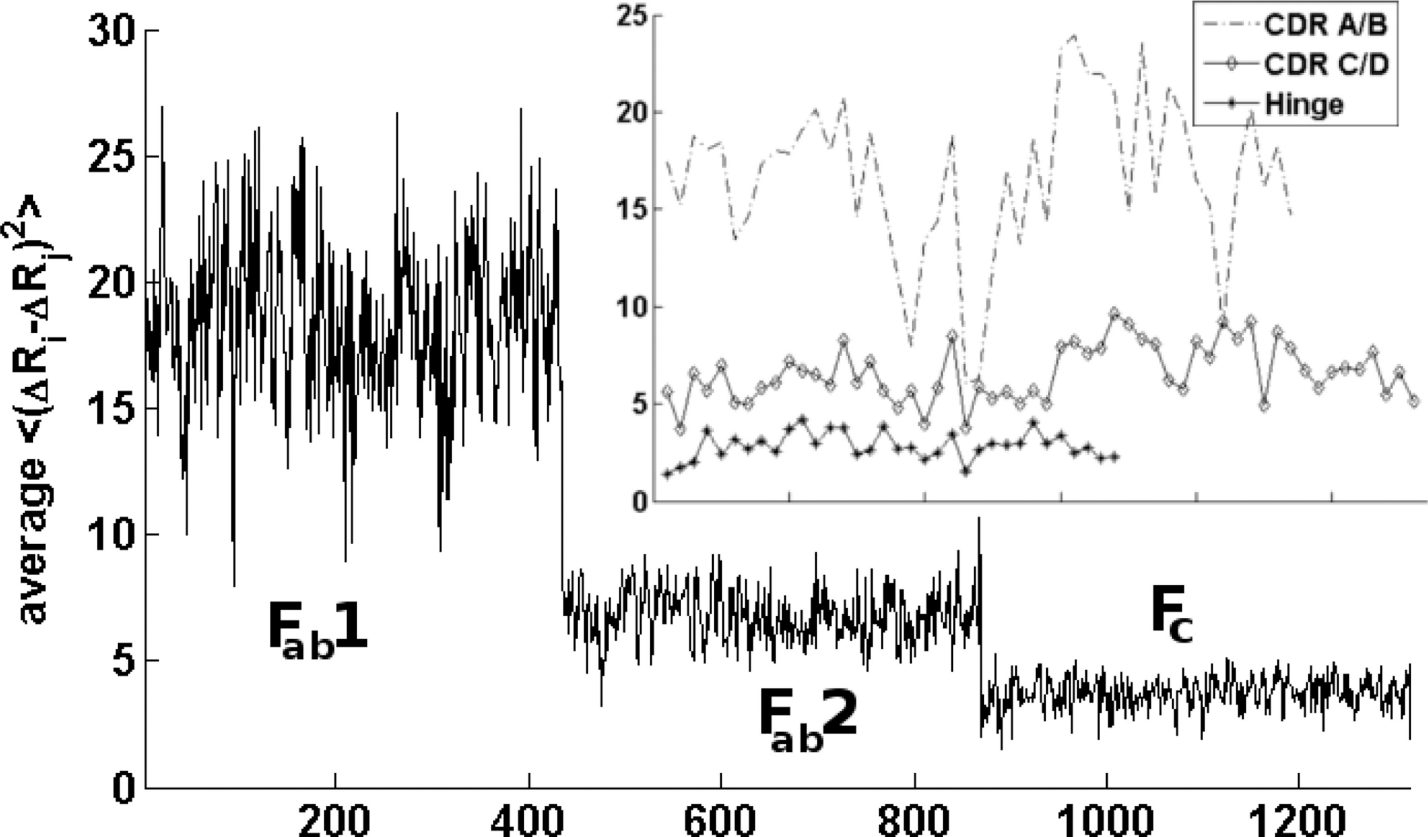
Mean-square internal distance changes within the entire immunoglobulin. (Inset) The CDRs from Fab1 are comprised of the variable loops in chains A and B, and the Fab2 CDRs are in chain C and D of 1IGT. We find that F_ab_ domains experience more internal motion than the F_c_, but that these are not symmetric. The inset shows the same quantity but specifically for the two CDR regions and the major hinge.

Another quantity that is informative about internal conformational changes is the mean-square internal distance (**MSID**) changes, <(Δ**R**_*i*_–Δ**R**_*j*_)^2^ >, given by equation 5. MSID changes can be calculated directly from the Hessian matrix that is used to generate normal modes in ANM with equation 6. This quantity describes the changes within a structure; how the normal modes stretch, compress, or otherwise alter the pair-wise distances between points in the structure. If this change in internal distance is zero for a given (i,j) pair, then the two points move together rigidly (the distance between them remaining unchanged). We have analyzed structures and seen that (data not shown) the areas of a protein with the smallest internal mean square distance changes are the cores of domains with these values increasing further away from stable cores. We have employed ANM models built with uniform springs with cutoffs ranging from 10–15 Å and with springs having inverse square dependences on distance. All of these yield similar results. Figure 5 shows this quantity averaged across all pairs of points within 7Å of one another. The CDR and major hinge are shown separately. We see that the CDR and hinge regions do not have significantly lower or higher average RMSID. We find that the F_ab_ domains experience more internal motion than the F_c_, but that the two F_ab_s are not symmetric in their motions. This is likely due to the asymmetry in the initial structure. But, other feasible structures might be expected to actually behave in a symmetric way. F_ab_2 is closer to the F_c_ than F_ab_1 and has more connections (higher stiffness) with it. Notably, we find that the internal distance changes at the hinge, as usual, are relatively small.

**Figure 6:**
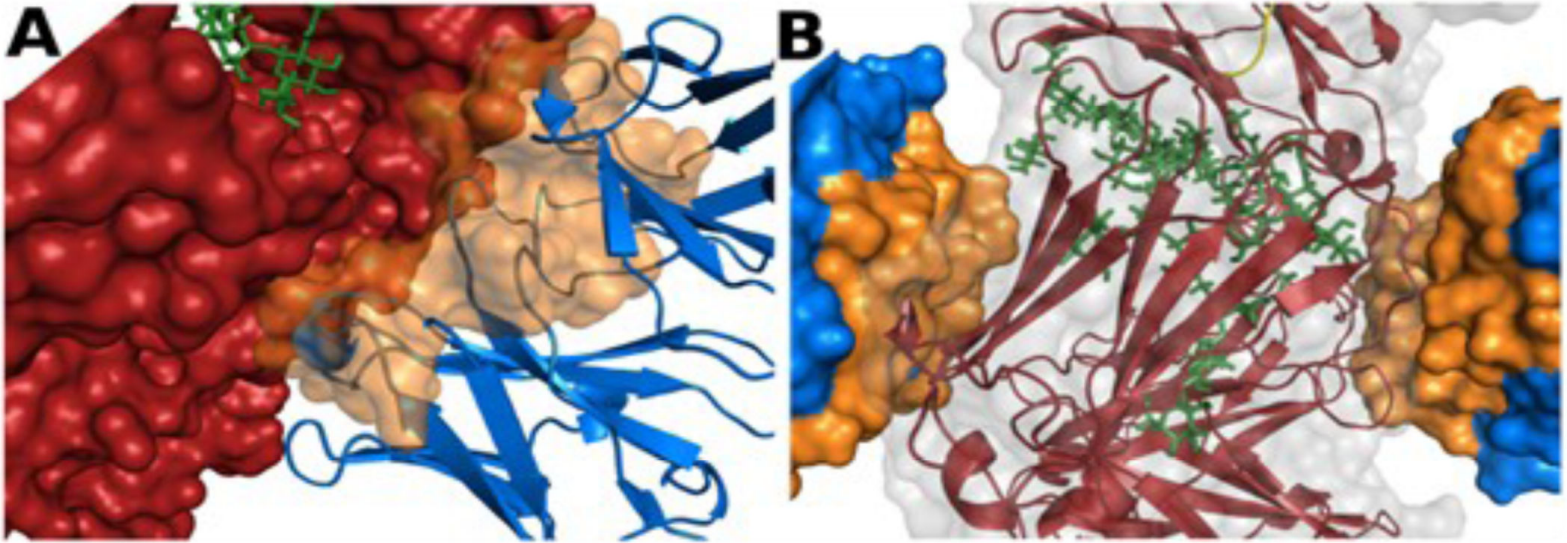
Intermolecular crystal packing in 1IGT. (A) One F_ab_ domain in 1IGT is shown in blue with its CDR as an orange surface. A symmetry related IgG is interacting with this CDR in the protein crystal. It is shown as a red surface and green sticks for the bound N-Acetyl-D-Glucosamine. (B) The F_c_ domain of 1IGT is shown as a red cartoon with gray transparent surface. Two intermolecular interacting F_ab_s are shown in blue whose CDRs are highlighted in orange. These two views highlight crystal packing via CDRs contacting F_c_.

For many proteins, it is common to compare motions from the computations with the crystallographic temperature factors, the B-factors. The B-factors describe the uncertainty assigned to a given atom, usually by assuming it originates from relevant internal fluctuations. Rigid body contributions are often removed by the crystallographer, but the successes of the TLS [26,27] and vGNM [28] methods provide strong evidence that B-factors often contain significant rigid body contributions. In the case of the 1IGT crystal we find that the B-factors may not be representative of the solution dynamics since the CDR of each F_ab_ is strongly bound to the F_c_ of another IgG (see Figure 6). While the experimental B-factors do highlight the major hinge as the most flexible part of the structure it is important to note that this is not because it is allowed to flex in the protein crystal, since the molecules are highly restrained by intermolecular interactions.

Intrinsic disorder in proteins is a topic of growing popularity. Two disorder predictors were applied to the IgG structure, DisEMBL [29] and POODLE [30]. Interestingly, both methods predict the most mobile part of the structure, the CDR, to be the least likely to be disordered. Both methods have components in their scoring scheme that are knowledge-based; learned from scanning the PDB. We believe the CDR is predicted to be so stable because of the abundance of IgG structures and because the CDR is rarely unbound. Because the CDR is almost never free to move, it is always ordered in the known structures. DisEMBL predicts disorder while the POODLE prediction predicts 3 quantities; the secondary structure as coil, the residues un-resolved in a crystal, and the probability of residues having a high B-factor. Both methods employ a probability cutoff of 0.5; any residue above the threshold is deemed to be disordered. Interestingly, the CDR is predicted to be the least disordered part of the structure. It is possible, due to the ambiguity that remains in defining protein disorder and the complexity of crystal B-factors, that some knowledge based disorder predictions may not be predicting exactly what one expects.

**Figure 7:**
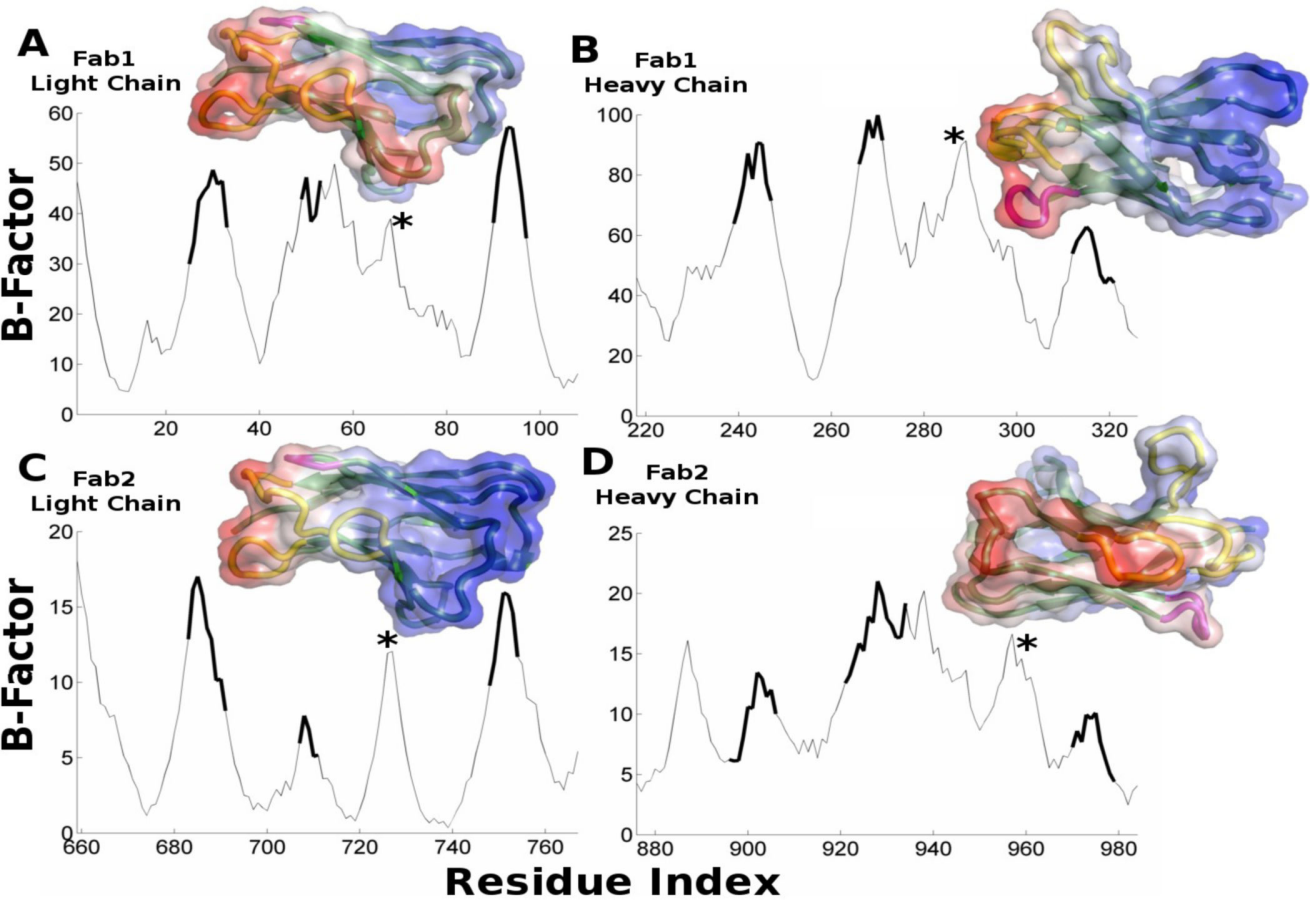
The computed mean-square fluctuations in positions of the F_ab_ residues, with the CDR residues highlighted with a thicker line. Molecular structures are shown with a semi-transparent surface colored blue to red for low and high computed B-Factors, respectively. Each plot has 4 peaks. Three of these correspond to the CDR and are colored yellow while the fourth is marked with an asterisk and colored purple. The remainder of the structure is colored green. In A, B, and C the CDR faces to the left while in D it faces right. This has been done because in D the back side is less mobile and the yellow CDR loops are less distinguishable when looking through the blue surface. In the F_ab_-like T-cell receptor the fourth loop (*) is also variable in sequence.

In Figure 7 we show the mean-square fluctuations of the IgG variable fragments computed with the ANM model. We find the parts of the structure that are most variable in sequence, the CDRs, are also the most mobile. It is interesting to note that ANM indicates 4 loops with high spatial mobility (and also the N-terminus), but there are only 3 CDR loops in the variable domain of each chain. The fourth loop is colored purple in the inset molecular images in Figure 7. Each F_ab_ domain then has two of these conserved mobile loops with one on either side of the CDR. It is interesting to note that the F_ab_-like T-cell receptor (TCR) has the same spatial arrangement of loops, but the fourth loop found to be mobile here is also hypervariable in sequence [31]. While this loop does not usually bind antigen, it is involved in nonspecific antigen binding of TCRs.

**Figure 8:**
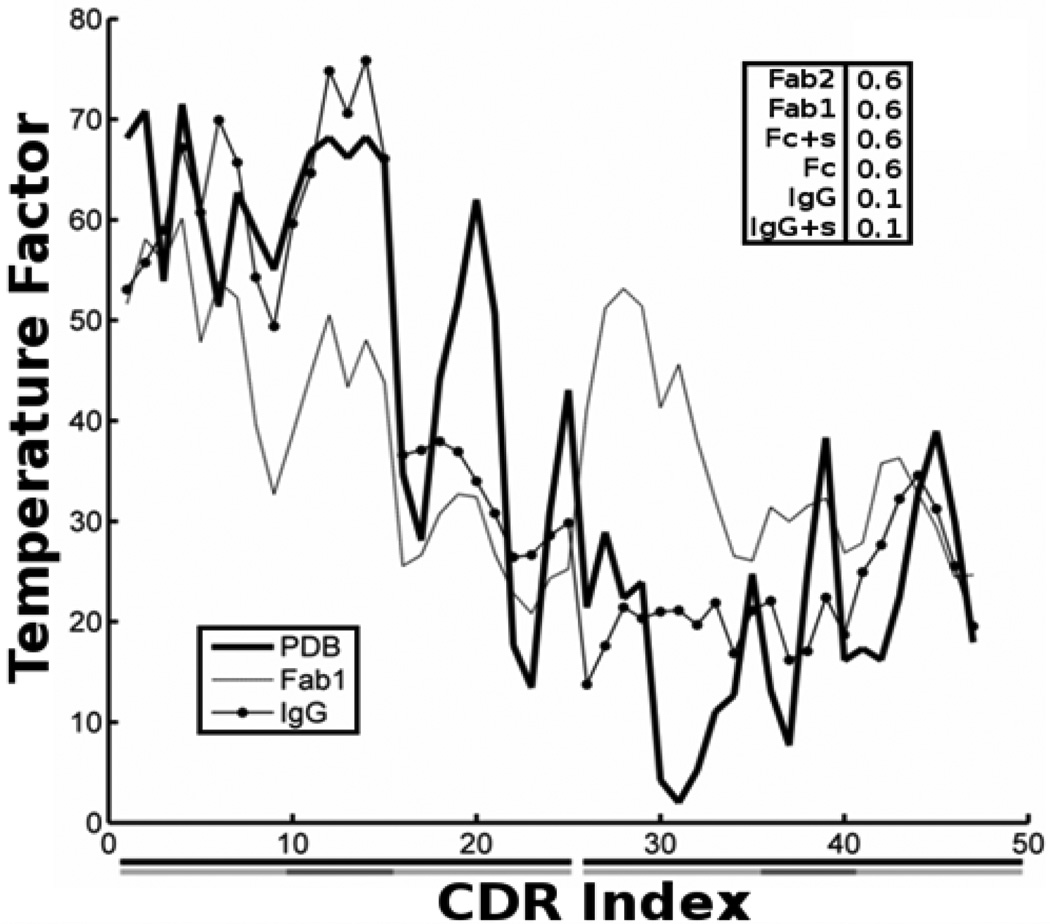
Correlation of experimental and computed temperature factors of the CDRs is stronger when the full IgG structure is considered than when parts of the structure are considered. Experimental temperature factors from the CDR are plotted in the heavy line. The first 25 residues plotted are from the heavy chain and the next 22 from the light. Heavy (left) and light (right) chains are delineated by black bars underneath the residue index and individual CDR hypervariable loops are shown by gray bars. CDR motion computed from the full IgG has a higher correlation (0.87) with experimental data than motion computed for the CDR using only the F_ab_ domain (0.50) when the 50 lowest frequency normal modes are used. The correlation between the two theoretical curves is 0.60. (inset) The correlations between temperature factors from the experimental B factors and computed from GNM models are shown. The magnitude of the correlation is not significantly affected by the choice of cutoff value. “+s” indicates the inclusion of coarse grained points from sugar molecules that attached to the F_c_. It is evident that the motions available to IgG in the crystal environment are not likely to be identical to those in solution.

**Figure 9:**
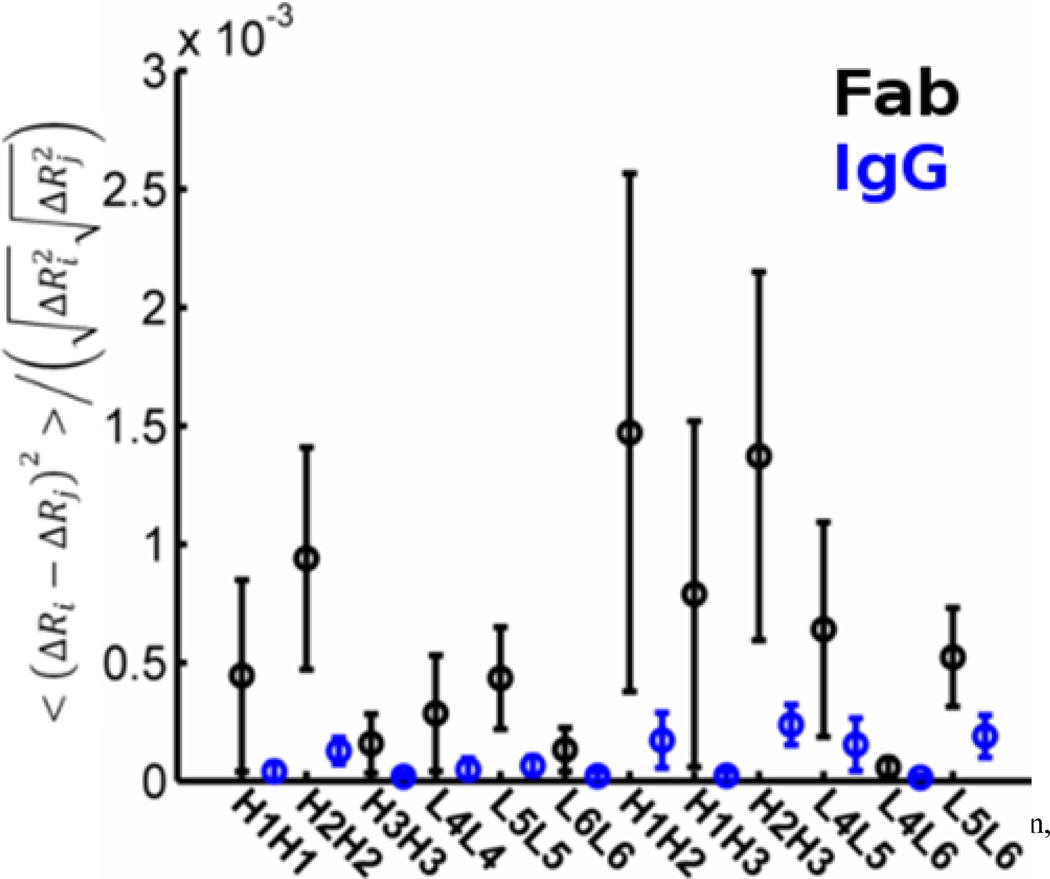
Inclusion of the full IgG structure diminishes the magnitude of internal distance changes. Normalized internal distance changes within the CDR calculated from ENM modes are scaled according to equipartition and constructed from the full IgG structure (blue) and from a single Fab domain (black). The IgG structure it-self appears to facilitate significantly larger excursions of the CDR loops away from their native positions, but does so without any significant internal rearrangements. The non-normalized changes are significantly greater for IgG.

To further investigate the motion patterns within the CDR and whether these may indicate that the IgG structure it-self facilitates excursions of the hypervariable loops, we consider the difference in the mean square fluctuations, internal distance changes, as well as spectral dimensions and fractal dimensions of the full IgG structure and of the individual F_ab_ domains. GNM usually predicts the mean square fluctuations of each point more accurately (as judged with crystallographic B-factors) than ANM. Motions of the CDRs from the GNM are shown in figure 8. Curves from the full IgG structure and for the F_ab_1 domain have been scaled overall to match the range of the crystal B-factors. Including the the whole structure yields a correlation with experiment of 0.87 for the F_ab_1, whereas utilizing only the structure of the F_ab_1 for the computations yields a correlation of 0.5. We have previously noted that the B-factors in this structure have the F_ab_ locked in a bound state. Interestingly, the computed CDR motion appears to be captured better by the full IgG model than by use of the Fab only. Internal distance changes, computed by using equation 5, indicate the extent of deformation between pairs of points in the structure. In figure 9, the effect of the full structure on the magnitude of change within and between hypervariable loops is summarized. The mean change within a (or between) loop(s) is plotted with error bars indicating one standard deviation. Before computing the extent of fluctuation, we rescale each mode to agree with equipartition wherein each internal degree of freedom would be assigned energy [32]. The full IgG structure amplifies internal distance changes within and between hypervariable loops, relative to a single F_ab_. This is further indication that the whole antibody structure may facilitate CDR configurational sampling so that a proper binding pose is found.

**Figure 10:**
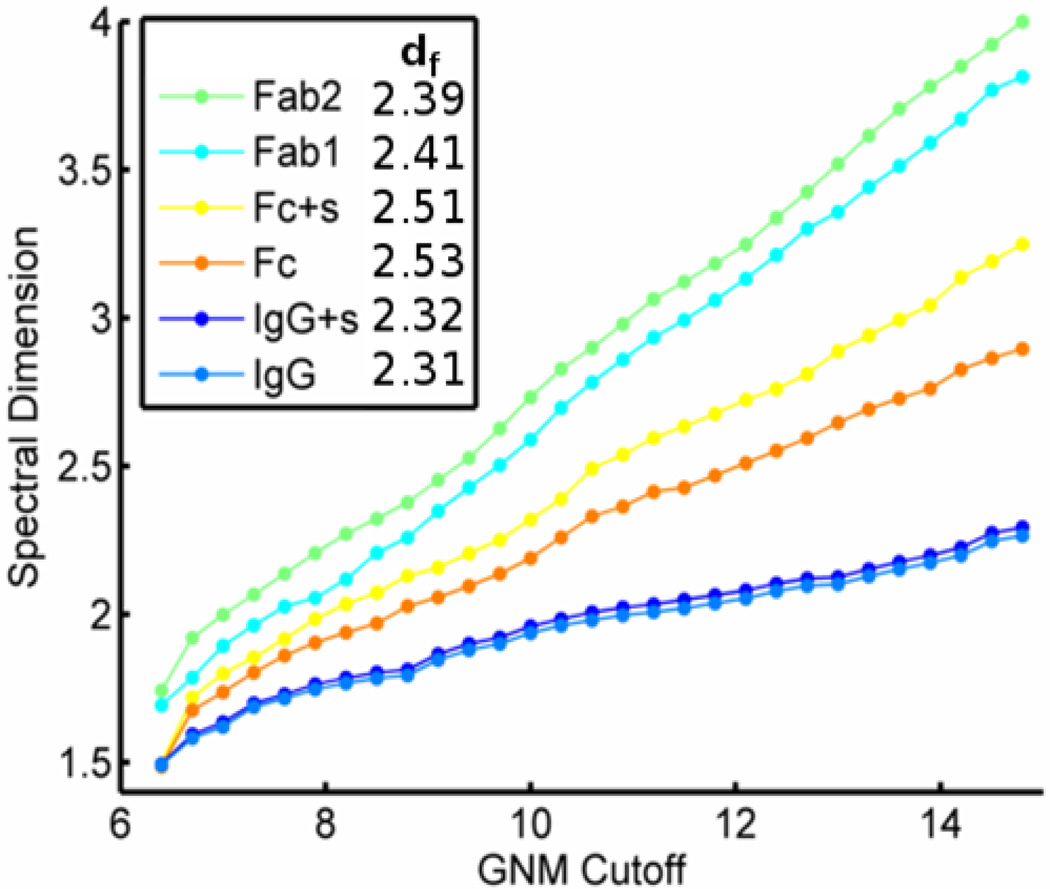
Spectral and Fractal dimension of IgG and its domains. The full length IgG has a lower spectral dimension no matter the cutoff employed. For the typical GNM of 7.3 the spectral dimension of IgG is 1.7. This is in good agreement with experimental measures on other proteins. The Fab domain alone has a larger spectral dimension of about 1.9, but all structures considered have a similar fractal dimension. “+s” indicates the inclusion of coarse-grained points from sugar molecules that are affixed to F_c_.

Information transfer within molecular structures has been the focus of numerous studies including the consideration of protein structures being fractal in nature (see Methods). Following previously established algorithms, we compute the fractal and spectral dimension of IgG and single F_ab_ domains (Figure 10). Briefly, the fractal dimension describes how the mass captured by concentric spheres scales with the radius of these spheres, and the spectral dimension describes how the frequencies of vibration for the structure scale with the density of modes. We find that the full length IgG behaves nearly like a 2D object in terms of its ability to transfer information from one part of the structure to another. Information transfer is thus significantly faster than one would expect from a uniform crystal lattice. This spectral dimension is in the range expected for proteins [24]. Interestingly, we find that the full structure has a lower spectral dimension than any single domain, again pointing to the possible utility of the whole structure for finding the right binding pose.

## Conclusion

Normal mode analysis using ANM are shown here to detect the hinge motions within the dominant low frequency motions, as well as the internal motions of the IgG structure. We have also evaluated the protein crystal and compared against the experimental temperature factors and disorder predictions. We find that the protein environment may be misleading in the crystal regarding the actual functional motions. Crystallographic temperature factors also reflect the crystal intermolecular interactions, which are extensive in this structure. Modeling approaches such as those applied here can provide a more comprehensive view of the biomolecule and its functional motions. The spectral dimension relates the density of vibrational modes to their frequency and can be used as a judge of the efficiency of energy transfer through a structure. Since this quantity is lower for IgG than for any individual domain, the hypothesis that hinge motions (the dominant computed motions) facilitate CDR motion is strongly supported. We conclude that the IgG structure is specifically designed to facilitate large excursions of the F_ab_ domains, as shown with the present methods for computational evaluation of the extent of hinge motions and the spatial sampling by components of the structure. Normal modes derived from the simplest potential function afford a good approximation to the total hinge motion and predict the most sequence-variable regions also to be the most spatially mobile – facilitating the binding of the F_ab_s. Our results may impact immunology by suggesting ways to include flexibility in the docking to predict the bound structures of IgGs.

## Figures and Tables

**Figure 1:**
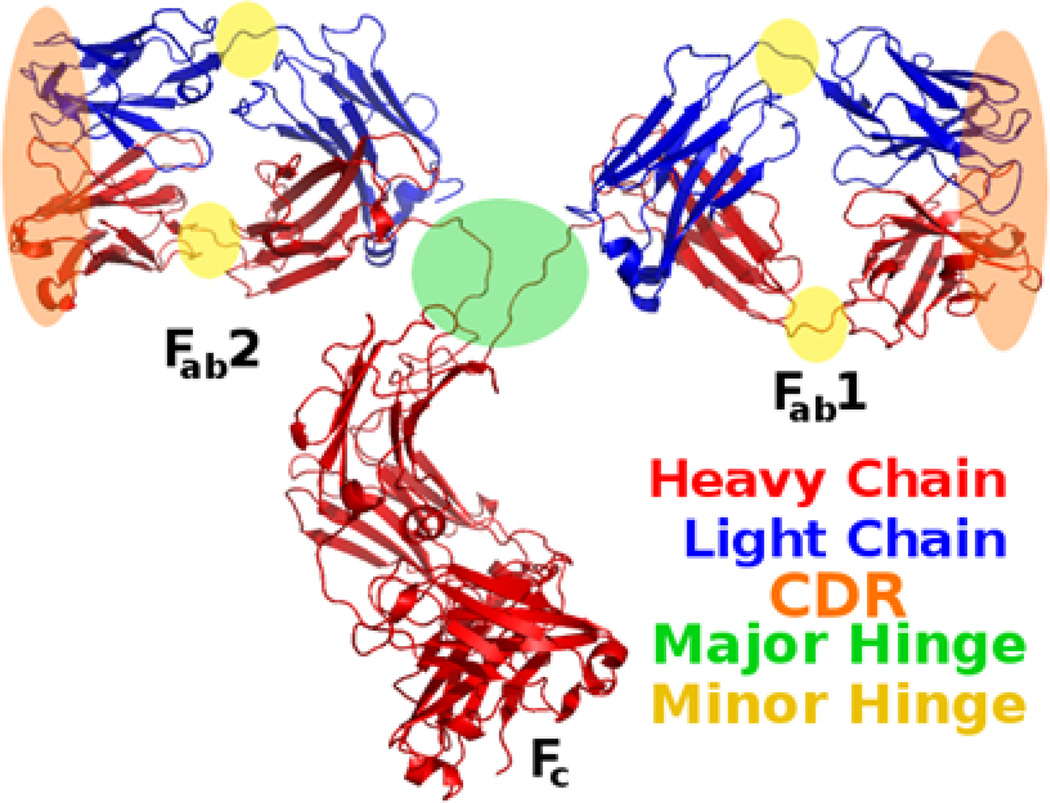
The structure of IgG (PDB structure 1IGT). Heavy and light chains are distinguished from one another by colors. The F_ab_ domains have orange ellipses indicating the locations of the hypervariable loops (the CDRs) and green and yellow circles identifying the major and minor hinges. F_ab_ domains consist of one light chain and half of a heavy chain and are connected to the F_c_ and each other by the major hinge.
